# Stability of salivary microRNA measures across an NCAA Division I football season: Implications for microRNA as a biomarker of concussion

**DOI:** 10.1371/journal.pone.0339614

**Published:** 2026-01-08

**Authors:** Thomas R. Campbell, Martina Zamponi, Delaney Leathers, Julie Cavallario, Jessica C. Martinez, Peter A. Mollica

**Affiliations:** 1 School of Rehabilitation Sciences, Ellmer College of Health Sciences, Old Dominion University, Norfolk, Virginia, United States of America; 2 Macon and Joan Brock Virginia Health Sciences, Eastern Virginia Medical School at Old Dominion University, Norfolk, Virginia, United States of America; 3 School of Medical Diagnostic and Translational Sciences, Ellmer College of Health Sciences, Old Dominion University, Norfolk, Virginia, United States of America; Sheffield Hallam University, UNITED KINGDOM OF GREAT BRITAIN AND NORTHERN IRELAND

## Abstract

**Background:**

Clinicians often face challenges in concussion care due to a heavy reliance on subjective patient input. Recently, research has sought objective biomarkers, like salivary microRNAs, to improve concussion management. However, significant limitations hinder the use of microRNAs as a diagnostic tool, including the cumulative effects of a contact sport season. A better understanding of the response to a contact sport season would help researchers and clinicians interpret expression changes at the time of injury in the context of seasonal variation. in Therefore, this study investigated the reliability of previously identified salivary microRNA targets across one contact sport season.

**Methods:**

This longitudinal study involved 50 male NCAA Division I football players (21 ± 1.6 years; 187.5 ± 6.9 cm; 103.1 ± 19.8 kg). Saliva was collected before the season’s first contact practice and within 72 hours of the season’s final game. Quantitative polymerase chain reaction (qPCR) experiments were conducted using pre-selected microRNA targets. Non-parametric tests compared expressions between time points (α ≤ 0.05).

**Results:**

No significant differences were found between pre- and post-season miRNA (*p* = .07−.46). However, intraclass correlation coefficients revealed low to moderate reliability across the season (ICC = −.04−.65).

**Conclusions:**

Our study found no significant differences in time points for target microRNA, but ICC statistics indicated low reliability across the season. These findings suggest that microRNA expression may be variable throughout the season regardless of concussive trauma, and clinicians should be aware that changes in microRNA expression should not be directly attributed to concussive forces. Researchers and clinicians should not rely on the presented set of microRNA to make clinical decisions for potential concussive injuries.

## Introduction

More than 4 million concussions are sustained in the United States annually, many occurring within athletic settings [1,2]. Mounting evidence suggests that this figure likely underestimates the actual burden of concussions due to underreporting, inadequate recognition of symptoms, and the limitations of current diagnostic tools [3–9]. Many athletes may fail to self-report symptoms due to a desire to continue participating or a lack of understanding of the severity of concussions. In contrast, others may have symptoms that go unrecognized by clinicians [3,4]. Current objective diagnostic measures are limited by concerns regarding their reliability, sensitivity, and specificity, particularly in the acute phase post-injury. This has generated concerns regarding their clinical utility for effectively identifying and managing concussions, leading to potential missed diagnoses [3–9].

Given the diagnostic challenges and the increasing awareness of the long-term neurological sequelae associated with repeated concussions, including early-onset Alzheimer’s disease and other neurodegenerative conditions, it is imperative that more reliable and valid objective measures be identified. One potential measure could be a biological indicator, or biomarker [10–13]. The discovery of such biomarkers could revolutionize concussion diagnosis and management, leading to more accurate and early identification of concussions, thereby allowing for timely interventions that may mitigate the risk of chronic neurodegenerative consequences.

Emerging research has highlighted salivary microRNA (miRNA) as a promising candidate for the non-invasive detection of concussions. MiRNAs are small, non-coding RNA molecules regulating gene expression at the post-transcriptional level, influencing various cellular processes [14–17]. These molecules are found in multiple biofluids, such as serum, plasma, cerebrospinal fluid, and saliva, and are considered highly stable under various physiological conditions [14–17]. Their stability and the non-invasive nature of saliva collection make them an attractive target for sideline diagnostic assessments in sports medicine. Moreover, miRNAs can be rapidly released into the oropharynx by cranial nerves (V, VII, IX, X, XII) following head trauma, making them an early indicator of concussion [14].

Recent advances in miRNA profiling have revealed the presence of over 2000 miRNAs in humans, with approximately 70% expressed in the central nervous system (CNS), underscoring their potential relevance as biomarkers for neurotrauma [14,18]. Certain miRNAs are specifically observed with pathophysiological changes after concussion, identifying them as potential diagnostic tools. Current literature identifies approximately 290 miRNAs associated with concussion spanning multiple biofluids, including cerebrospinal fluid, blood, and saliva [15–23]. Of note are 49 miRNAs identified in saliva that exhibit potential as biomarkers for concussion, with 21 linked to concussion management and 34 associated with concussion diagnosis [15–23]. These findings have established a foundation for further investigation into the utility of salivary miRNA in concussion diagnostics. Our previous work synthesized the literature on salivary miRNA as a concussion biomarker and identified six target miRNAs as ideal candidates for evaluation in the current study [24].

Despite the promising potential of salivary miRNAs, several gaps in the literature still need to be addressed. One critical limitation is the need for longitudinal data examining the stability of miRNA expression profiles over time, particularly in the context of repeated impacts, common in collision sports like football. Determining whether miRNA expression remains stable throughout a sports season without clinically diagnosed concussions is necessary. Such data would help delineate injury-specific changes from fluctuations in miRNA expression due to time.

This study aimed to examine the effects of one NCAA Division I football season on salivary miRNA expression. We hypothesized that there would be no statistically significant differences between pre- and post-season expressions of the pre-selected miRNA.

## Materials and methods

Ethical approval for this study was granted by the University’s Institutional Review Board (IRB#1563355). All participants provided written informed consent before participation by the Declaration of Helsinki.

### Design and participants

This prospective, observational study employed a case-study design involving a convenience sample of 50 NCAA Division I football players from one institution, all medically cleared for participation in intercollegiate athletics by the institution’s sports medicine staff consistent with the institution’s policy and practice for all intercollegiate athletes. Medical clearance included a thorough review of medical history, musculoskeletal assessment, neurological assessment, and evaluation of the current overall condition health. Participant enrollment occurred between July 2021 and December 2021. Exclusion criteria included being currently concussed at the time of enrollment or any significant neurological disorders (e.g., seizure disorder, Guillain-Barre syndrome, stroke).

### Demographics and medical history

Participants self-reported demographic and medical history data, including age, sex, playing position, and significant neurological disorders. Additionally, participants reported concussion history data, including the number of diagnosed concussions, most recent concussion, and time taken to full recovery following injury.

### Saliva sample collection

The principal investigator collected all saliva samples using ORAcollect-RNA ORE-100 swab kits (DNA Genotek, Ottawa, Ontario, Canada). Per manufacturer’s guidelines, collection involved swabbing between the participant’s cheek and gums on the lower jaw while minimizing contact with teeth. After swabbing, the sample was placed into a storage tube and shaken vigorously fifteen times. Samples were collected following participants performing an oral tap water rinse 15 minutes beforehand [22,25]. Saliva samples were stored at −80°C until processing.

### Fatigue Assessment Scale (FAS)

The Fatigue Assessment Scale [26–28] (FAS) was used to assess individuals’ change in fatigue during one NCAA Division I football season. The FAS consists of 10 items rated on a 5-point Likert scale (1 = never, 5 = always) and provides an overall fatigue score ranging from 10 (never fatigued) to 50 (always fatigued). Participants completed the FAS pre-season and post-season and were asked to describe how they felt over the past seven days to track any changes in fatigue throughout the season. Given the known diurnal oscillations in salivary miRNA expressions, this assessment was intended to mitigate potential circadian influences on biomarker variability [29].

### Timing of data collection

Baseline data collection occurred one week before the first structured pre-season practice. Participants completed a demographics questionnaire, medical history questionnaire, and the FAS before providing saliva samples. After the football season, all participants returned to the athletic training clinic within 72 hours of the final competition for post-season assessments, including saliva sample collection, medical history updates, and FAS completion.

### Saliva sample processing

Total RNA was isolated from each saliva sample per manufacturer protocol using the miRNeasy Kit (Qiagen, Inc., Germantown, MD, USA). RNA quality and quantity were assessed using a Thermo Scientific Nanodrop 2000 Spectrophotometer (Thermo Fisher Scientific, Wilmington, DE, USA) and a Thermo Fisher Qubit 4 Fluorometer (Thermo Fisher Scientific, Wilmington, DE, USA). miRNA was extracted using the MagMAX mirVana Total RNA Isolation kit (Thermo Fisher Scientific, Wilmington, DE, USA) and processed on a KingFisher Flex Magnetic Particle Processor (Thermo Fisher Scientific, Wilmington, DE, USA). Complementary DNA (cDNA) synthesis was performed using a TaqMan® Advanced miRNA cDNA synthesis kit (Thermo Fisher Scientific, Wilmington, DE, USA). Quantitative real-time polymerase chain reaction (qPCR) was conducted on a StepOnePlus Real-Time PCR System (Applied Biosystems, Waltham, MA, USA), using specific TaqMan advanced miRNA assays for the following target miRNAs: miR-29c-3p, miR-26b-3p, miR-192-5p, miR-27a-5p, miR-30e-5p, miR-7–1-3p, miR-361-5p (endogenous control). PCR conditions were standardized: 95.0°C for 20 seconds, then forty cycles of 95.0°C for 1 second and 60°C for 20 seconds.

### Statistical analysis

Expression fold changes in miRNA were calculated using the 2^-ΔΔCT^ method, with miR-361-5p as the endogenous control [30]. Each target miRNA underwent three PCR replicates per sample, and the mean cycle threshold (CT) was calculated. The ΔCT (change in CT) was determined by subtracting the mean CT of the endogenous control from the mean CT of each target miRNA. For non-baseline samples, ΔΔCT values were calculated by subtracting the ΔCT of subsequent samples from the baseline ΔCT for each target miRNA. Fold changes in miRNA expression were then calculated using the formula 2^-ΔΔCT^ [31].

Paired t-tests were employed to compare pre- and post-season FAS scores. Intraclass correlation coefficients (ICC) were calculated to assess the consistency between pre- and post-season saliva samples. Estimates of ICC and their 95% confidence intervals were calculated using IBM SPSS Statistics (IBM Corporation, version 28.0, Armonk, NY, USA) based on an absolute-agreement, 2-way mixed-effects model. For this study, any ICC value below 0.5 was regarded as low reliability; 0.5–0.75 as moderate reliability; 0.75–0.9 as good reliability; and above 0.9 as excellent reliability [32]. Non-parametric tests (Wilcoxon signed-rank test) were used to compare ΔCT values across time points (pre-season vs. post-season). Effect sizes were interpreted as low (0.10), medium (0.30), and large (0.5) [33]. All statistical significance was set a priori at *p* < 0.05.

## Results

### Participant characteristics

Fifty male collegiate athletes aged 18–24 (*M* = 21 ± 1.6 years) participated in the study, Table 1. At pre-season data collection, 34% of the participants reported a previous history of concussion. One participant did not complete post-season data collection due to a season-ending injury and a subsequent temporary withdrawal from the university.

**Table 1:** All participant demographic information.

	Pre-Season (*n* = 50)	Post-Season (*n* = 49)
Demographics		
Age (year)	21 ± 1.64	
Height (cm)	187.45 ± 6.99	
Weight (kg)	103.14 + 19.83	
Medical History		
Seizure Disorder	0	0
Guillain-Barre Syndrome	0	0
Stroke	0	0
Head Injury Details		
No. Participants with Previous Concussions	17	20
Mean Previous Concussions Dx (Concussion Hx only)	1.47 ± 0.94	1.40 ± 0.60
Mean Previous Concussions Un-Dx (Concussion Hx only)	0.19 ± 0.54	0.00 ± 0.00
Fatigue Assessment Scale		
Composite Score	18.96 ± 4.11	18.67 ± 4.10

### Fatigue assessment scale (FAS)

No statistically significant differences were observed in FAS scores between pre-season (*M* = 18.94, *SD* = 4.15) and post-season (*M* = 18.67, *SD* = 4.09), *t*(48) =.409, *p* = .684, indicating no meaningful change in fatigue levels experienced over the season.

### Effects of one NCAA division I football season

Salivary miRNA stability across the season was assessed by comparing pre- and post-season measures of multiple miRNA targets. Overall, low reliability was observed for most miRNA, with only miR-29c-3p and miR-27a-5p demonstrating moderate reliability. Specifically, miR-29c-3p had an intraclass correlation coefficient (ICC) of 0.52 (*F*(49, 49) = 2.09, *p* < 0.01), while miR-27a-5p had an ICC of 0.65 (*F*(49,49) = 2.89, *p* < 0.01). In contrast, other miRNA targets showed lower ICCs, with values ranging from 0.21 for miR-26b-3p to −0.16 for miR-7–1-3p, all demonstrating low reliability (ICC < 0.5), Table 2 [32].

**Table 2:** Effects of one NCAA Division I football season on target microRNA.

miRNA	ICC	95% CI Lower Bound	95% CI Upper Bound	Pre-season Median (IQR)	Post-season Median (IQR)	*z*	*p*-value
miR-29c-3p	0.52	0.16	0.73	−3.58 (1.13)	4.19 (1.01)	−0.95	0.34
miR-26b-3p	0.21	−0.42	0.55	−0.08 (2.16)	1.79 (1.68)	−0.74	0.46
miR-192-5p	0.19	−0.43	0.54	−2.50 (1.94)	−0.96 (2.00)	−1.82	0.07
miR-27a-5p	0.65	0.39	0.80	−2.50 (1.76)	2.57 (1.91)	−1.09	0.28
miR-30c-5p	−0.04	−0.78	0.40	−0.54 (1.53)	−0.95 (1.16)	−1.04	0.30
miR-7–1-3p	−0.16	−0.94	0.34	1.26 (0.85)	1.36 (0.62)	−1.02	0.31

**Fig 1:**
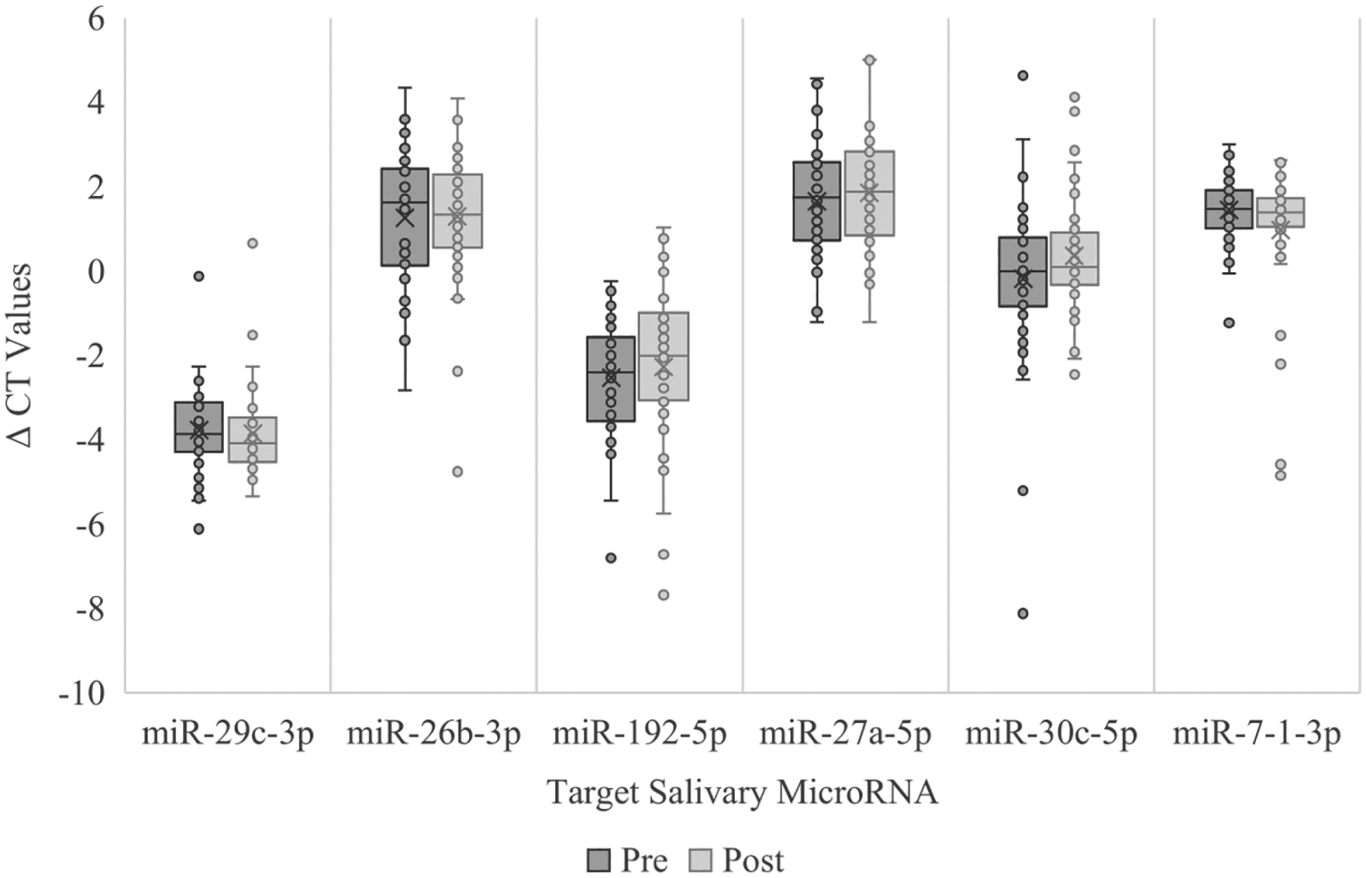
Season effects on target salivary miRNA ΔCT Values.

Comparisons of pre- and post-season salivary miRNA expression levels showed no significant differences for target miRNAs. For miR-29c-3p, pre-season levels (*Mdn = −3.58*) did not significantly differ from post-season levels (*Mdn = −4.19*), *z* = −0.95, *p* = 0.34. Similarly, non-significant results were observed for miR-26b-3p (*z* = −0.74, *p* = 0.46), miR-192-5p (*z* = −1.82, *p* = 0.07), miR-27a-5p (*z* = −1.09, *p* = 0.28), miR-30c-5p (*z* = −1.04, *p* = 0.30), and miR-7–1-3p (*z* = −1.02, *p* = 0.31). The effect sizes were small for all miRNA targets (*r* values ranged from −0.10 to 0.26), suggesting that one NCAA Division I football season did not significantly alter salivary miRNA expression, Fig 1.

## Discussion

**Fig 2:**
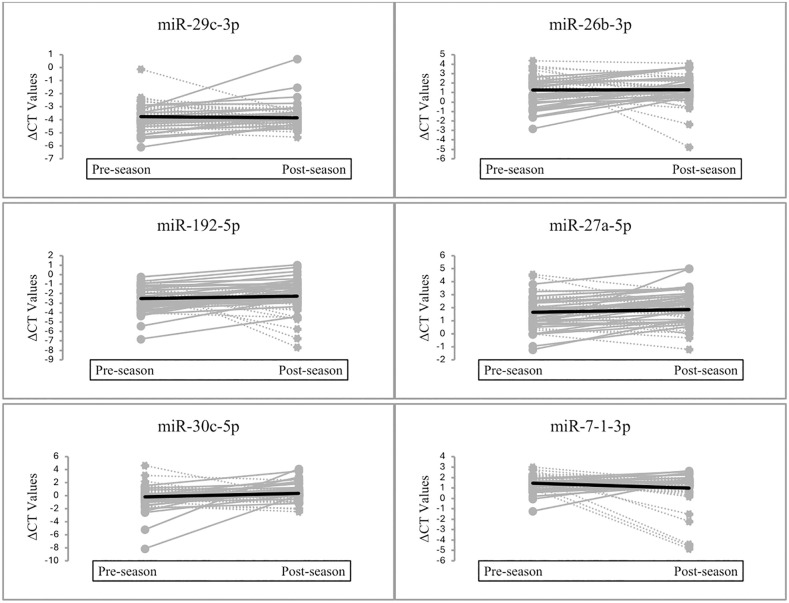
Paired pre- and post-season ΔCT Values for pre-selected microRNAs.

This study is among the first to longitudinally assess the effects of a single NCAA Division I football season on pre-selected salivary miRNA, offering a novel, non-invasive view of molecular changes linked to participating in a collision sport. We found that the pre-selected salivary miRNAs did not show a significant change in expression from pre-season to post-season, indicating that these miRNAs may not be impacted by participation in one collegiate football season. However, the ICC statistics for ΔCT values suggested low to moderate reliability for all target miRNA, indicating some variability in expressions across the season. Although group mean comparisons revealed no significant pre-to post-season differences in the pre-selected miRNA, individual-level analyses using ICC statistics demonstrated meaningful variability, where opposing expression patterns between athletes (e.g., high-to-low or low-to-high) effectively canceled out in the group mean, masking clinically relevant changes. Fig 2 shows individuals’ pre- and post-season expressions for each pre-selected miRNA and demonstrates the effects on group mean. This variability raises concerns about the stability of these biomarkers in the absence of a diagnosed concussive event. This variability underscores the need for caution in interpreting salivary miRNA stability, as expression appears to be dynamic, likely influenced by factors beyond just concussive injury, including physical exertion, inflammation, or exposure to repeated impacts as reported in prior biomarker studies [34,35]. As a result, it is challenging to conclude that changes in salivary miRNA expression observed at time of concussion are solely due to the injury itself. Although previous research has identified the miRNA targets evaluated in this study as potential biomarkers of concussion [15,21,25], the results do not fully support their use as a reliable clinical biomarker in their current form. Further research is needed to differentiate salivary microRNA expression patterns that arise specifically from concussive injuries versus those influenced by season-long factors such as exercise-induced inflammation or repeated sub-concussive impacts.

Our findings build on previous biomarker research in contact sports, which has primarily focused on blood-based protein biomarkers such as Tau, S100B, or neurofilament light chain [36,37]. Although these markers offer valuable insights into concussive injuries, they often require invasive sampling that is not easily performed on an athletics sideline and may miss subtle, cumulative changes that happen over a competitive season. In contrast, salivary miRNAs provide an objective, non-invasive alternative that might show immediate changes through expression via the cranial nerves [14,22]. Notably, the miRNA shown in this study displayed season-long changes, which aligns with earlier research linking miRNAs to head trauma [14–16,18,21–23,25,29]. However, the expression changes of individual miRNAs were not statistically significant with low reliability. This ultimately suggests that while patters of miRNA expression may reflect exposure across a season, the utility of any single miRNA as standalone biomarker is limited. By demonstrating changes in an NCAA Division I football season, our study provides necessary information on salivary miRNA as a biomarker for concussion. This fills a key gap between discovering new biomarkers and applying them in sports medicine practice.

To provide context for our findings, we analyzed the known biological roles of the six salivary miRNAs evaluated in this study. miR-29c-3p is brain-enriched and regulates extracellular matrix remodeling and neuroplasticity, with prior work linking its salivary expression to cumulative head-impact exposure in athletes [15,21,38]. miR-26b-3p modulates microglial inflammatory programming and has similarly been reported to vary with contact-sport impact load [21,39]. miR-192-5p is expressed in epithelial and endothelial tissues and regulates oxidative stress, apoptosis, and chemokine signaling, positioning it as a potential marker of systemic stress responses [40]. miR-27a-5p acts as a negative regulator of innate immune signaling by dampening TLR4/IRAK4-NF-κB pathways in microglia, while miR-30c-5p has been implicated in cardiovascular and metabolic regulation, including lipid metabolism and endothelial function, with emerging evidence for roles in stress and inflammation [41]. Finally, miR-7–1-3p, a member of the neuron-enriched miR-7 family, is known to repress α-synuclein and limit NLRP3 inflammasome activation, thereby contributing to neuroprotection [42,43]. Considering these roles, the absence of significant groupwise changes in our cohort does not contradict prior literature. miR-29c-3p and miR-26b-3p have been associated with exposure-dependent variability rather than uniform pre-/post-season shifts, while miR-27a-5p and miR-30c-5p may reflect stable homeostatic regulation [44]. The near-signal trend for miR-192-5p (*p* = 0.07) aligns with its context-dependent stress responsiveness, and low reliability of miR-7–1-3p may reflect instability in salivary detection rather than lack of biological relevance [42,43,45].

In summary, this study provides novel evidence that salivary miRNAs are sensitive to the cumulative demands of an NCAA Division I football season, limiting their individual potential as objective markers of concussion. By demonstrating longitudinal changes in the presented miRNA, our work highlights the need for additional research in this area. Future research should aim to enroll larger and more diverse cohorts, including female athletes and participants from different sports and levels of competition, and collect measures throughout the season to validate the use of salivary miRNA as a concussion biomarker. Incorporating tools such as accelerometers or video recordings to track head impacts could provide valuable insight into the effects of sub-concussive trauma on miRNA expression. Additionally, further studies should investigate the clinical utility of miRNA ratios and explore the potential for combining multiple biomarkers to improve diagnostic accuracy and specificity. Ultimately, these efforts could establish miRNAs as viable tools for concussion practices.

### Limitations

This study was not conducted without several limitations. First, the sample size was limited to all male participants from a single NCAA Division I football team, thus limiting the generalizability of the findings. Moreover, the study did not account for the number of sub-concussive head impacts sustained by each participant, which may have influenced miRNA expression over the season. Lastly, we did not control for demographic (e.g., age, race, etc.), health (e.g., concussion history, comorbidities, etc.), or sport-related (e.g., football position, number of games or practices, etc.) in the data that were collected. It is possible that these factors influence microRNA expression and should be considered as covariates in future studies of similar nature.

## Conclusions

This study aimed to examine the effects of one NCAA Division I football season on salivary miRNA expression. Our findings revealed no statistical differences between pre-season and post-season expressions of individual pre-selected miRNA, suggesting that participation in an entire NCAA Division I football season does not alter these markers. However, ICC values indicated low to moderate reliability, suggesting that natural variability in miRNA expression may occur throughout the season. The variability indicated through low to moderate ICC values could stem from inherent biological fluctuations or the modulation of miRNA expression that is stimulated by exertion, inflammatory activity, stress responses, or repetitive sub-concussive impacts, all of which are likely to have occurred during a football season. The variability of miRNA expression without a clear statistical change across individual miRNA complicates the interpretation of miRNA changes from purely injury-related causes. While our results do not support the immediate implementation of salivary miRNA biomarkers for concussion practices, they do provide important evidence of season variability and highlight the need for continued research to refine these markers, determine their reliability, and establish their specificity for future use in clinical application.

## Supporting information

S1 File Supporting data. All supporting data from pre- and post-season saliva collections.
(XLSX)
